# Moderate Alcohol Use and Mortality from Ischaemic Heart Disease: A Prospective Study in Older Chinese People

**DOI:** 10.1371/journal.pone.0002370

**Published:** 2008-06-04

**Authors:** C. Mary Schooling, Sun Wenjie, Sai Yin Ho, Wai Man Chan, May Ked Tham, Kin Sang Ho, Gabriel M. Leung, Tai Hing Lam

**Affiliations:** 1 Department of Community Medicine and School of Public Health, The University of Hong Kong, Hong Kong SAR, China; 2 Department of Health, The Government of the Hong Kong Special Administrative Region, Hong Kong SAR, China; Harvard School of Public Health, United States of America

## Abstract

**Background:**

Moderate alcohol use is generally associated with lower ischaemic heart disease (IHD) mortality but it is difficult to ascertain whether this is due to attributes of moderate alcohol users or the properties of alcohol itself. Evidence from populations with different patterns of alcohol use and IHD can provide crucial evidence. We assessed the association of moderate alcohol use with IHD mortality in older Chinese people from Hong Kong.

**Methodology:**

We used Cox regression to determine whether moderate alcohol use was associated with IHD mortality in a prospective, population-based cohort study of all 56167 attendees, aged 65 years or over, from July 1998 to December 2000 at all 18 Elderly Health Centers operated by the Department of Health in Hong Kong.

**Principal Findings:**

After a median follow-up of 4.2 years, there were 406 (188 in men, 218 in women) deaths from IHD in 54,090 subjects (96.3% successful follow-up). Moderate alcohol use in men was not associated with IHD mortality adjusted only for age [Hazard Ratio, HR 1.01 (95% CI 0.55 to 1.84) compared with never drinkers] or additionally adjusted for socio-economic status and lifestyle. Almost all women were occasional drinkers and their current alcohol use was not significantly associated with IHD mortality [HR 0.88, (95% CI 0.51 to 1.53)].

**Conclusions:**

Moderate alcohol use had no effect on IHD mortality in older Chinese men. Lack of replication of the usual protective effect of moderate alcohol use in a setting with a different pattern of alcohol use and IHD could be due to chance or could suggest that the protective effect of alcohol on IHD does not extend to all populations.

## Introduction

Many observational studies have shown that moderate alcohol use is associated with lower mortality from ischemic heart disease (IHD) [1]–[4]. Despite these findings, there are counter examples [5]–[8]. Observational studies, prospective or case-control, cannot distinguish whether the protection offered by alcohol is casual or due to moderate alcohol use acting as a marker for other mortality related factors, such as healthier behaviours [9], [10], more social integration [11] or better health status [12]. Many of these studies have come from western countries, where moderate alcohol use and lower risk of ischaemic heart disease may both be associated with higher socio-economic status [13]–[16]. Moreover, alcohol use is often a normal part of social interaction. Thus it is difficult to assess whether the apparent beneficial effect of moderate alcohol use on ischaemic heart disease mortality is real or the result of uncontrollable (or uncontrolled) residual confounding [17].

Evidence from settings with different social patterns of alcohol use and ischaemic heart disease can potentially make a contribution. Compared with many western countries, alcohol use is less prevalent and more restrained in southern Chinese, so the characteristics of moderate users may be different. In Hong Kong, only 55% of men and 19% of women drink alcohol with an average daily intake amongst alcohol users of 9 grams ethanol in men and 2 grams in women, and the main alcoholic beverage is beer [18]. In contrast, use of alcohol is more common in northern China, Japan and South Korea [19]–[21]. Moreover in Hong Kong ischemic heart disease is not clearly associated with lower socio-economic status although alcohol use may be [18], [22]. There have been few prospective studies of alcohol use and IHD mortality in Chinese [23], [24]. Both previous studies in men from Shanghai suggested that moderate alcohol use was associated with lower IHD mortality. Although, in both studies, moderate alcohol use had rather systematic effects, which suggests that some favorable characteristics of the moderate alcohol users may also have been playing a role. We took advantage of a large prospective cohort of older men and women to re-examine the relation between moderate alcohol use and IHD mortality.

## Methods

Details of the methods have been reported elsewhere [25]. Briefly, in July 1998, 18 Elderly Health Centers were established by the Department of Health, Government of HKSAR, to deliver health examinations and primary health care services for the elderly. All aged 65 years or older were eligible to enroll, and all those first enrolled from July 1998 to December 2000 were included in this study. More women enrolled than men; otherwise the subjects were similar in age, socio-economic status, current smoking and hospital use to the general elderly population [25].

### Alcohol use

Alcohol use was initially recorded in four categories i.e. never-drinker, ex-drinker, social drinker and regular drinker. Social drinkers are people do not usually drink alcohol, but who may drink alcohol at social gatherings on special festive occasions, such as at Chinese New Year or a wedding. Regular drinkers were further asked if they drank more than 3 units per day (men) or 2 units per day (women). A unit was defined as 10 grams of ethanol, and the interviewer was provided with a list for converting common alcoholic drinks into units, for example a 335 ml can of beer at 5% is 1.4 units. On this basis regular drinkers were categorized as moderate or excessive. Moderate drinkers were defined as regular drinkers of 3 or fewer units per day in men and 2 or fewer units per day in women. Excessive drinkers were defined as regular drinkers of more than 3 units per day in men and 2 units per day in women. Self-reports of alcohol use are prone to mis-reporting; as a check, we examined the association between alcohol use and esophageal cancer.

### Follow-up

Vital status was ascertained from death registration, special outpatient and hospitalization databases in Hong Kong by record linkage using the unique Hong Kong identity card number. The last date of follow-up or censor date was December 31, 2003. The subjects not found dead or alive by record linkage were followed up by telephone interview from November 2004 to January 2005, after which only 3.5% (1951) remained untraced. Adjusted for age, socio-economic status, lifestyle habits, untraced men were no different in alcohol use from traced men, but untraced women were significantly more likely to be social or excessive drinkers (odds ratio of being lost to follow-up 1.25 (95% CI 1.03 to 1.50) and 3.24 (95% CI 1.38 to 7.60) for social drinking and excessive drinking women respectively compared with never drinking women). Among the 54216 traced subjects, 3884 had died by December 31, 2003. Causes of death obtained for 3825 were routinely coded by the Department of Health according to the International Classification of Disease – 9^th^ Revisions before 2001 and 10^th^ Revision in and after 2001. Most Hong Kong residents die in hospital, enabling accurate ascertainment of cause of death, which we have used previously in similar studies [26].

### Outcomes

The primary outcome was cause-specific mortality from the IHD (ICD-9 410-4 or ICD-10 I20-5); a secondary outcome was cause specific mortality from cancer of the esophagus (ICD-9 150 or ICD10 C15).

### Statistical analysis

Chi-square tests were used to compare proportions of subject's characteristics across alcohol use groups. The Cox proportional hazards model was used to estimate the hazard ratios (HR) and the 95% confidence intervals (CI) for IHD and esophagus cancer mortality by alcohol use group adjusted in model 1 for age and in model 2 additionally for all other baseline potential confounders (education, monthly personal expenditure, housing type, exercise, body mass index (BMI) and smoking status), categorized as in Table 1. The proportional hazards assumption was checked by visual inspection of plots of log (- log (S)) against time, where S was the estimated survival function. Subjects who died of any other causes were regarded as censored at the date of death [27], [28]. Men and women were analyzed separately, because of the different patterns of alcohol use in men and women in Chinese society. Ethics approval was obtained from the Ethics Committee of the Faculty of Medicine, the University of Hong Kong. The study complied with the Declaration of Helsinki.

**Table 1:** Baseline characteristics of 54090 enrollees at Elderly Health Centres in Hong Kong from 1998 to 2000 with follow-up

		Men						Women					
		Alcohol use status						Alcohol use status					
		Never drinker (n = 9,177)	Ex-drinker (n = 3,300)	Social (n = 3,957)	Moderate (n = 1,318)	Excessive (n = 375)	χ^2^ p-value	Never drinker (n = 30,763)	Ex-drinker (n = 1,987)	Social (n = 2,856)	Moderate (n = 306)	Excessive (n = 51)	χ^2^ p-value
Age group	65-9	38.2	30.6	46.0	42.3	44.3		39.5	32.2	46.7	39.2	47.1	
	70-4	31.4	34.0	32.1	34.2	36.0		30.3	32.4	29.2	32.7	29.4	
	75-9	19.4	21.2	15.1	16.2	13.1		18.5	20.8	16.2	19.3	15.7	
	80-4	8.0	10.5	5.5	5.7	4.8		8.2	9.7	6.0	6.5	3.9	
	≥85	3.0	3.7	1.3	1.6	1.9	<0.001	3.6	5.0	1.9	2.3	3.9	<0.001
Education	Post-secondary	8.0	4.2	6.5	5.6	4.0		1.9	1.4	2.0	1.6	0.0	
	Secondary	25.7	18.0	24.7	21.1	17.3		8.1	4.3	8.0	9.2	7.8	
	Primary	49.9	55.0	53.1	53.7	54.1		29.4	25.5	30.2	27.1	29.4	
	No formal education but literate	9.5	13.0	9.2	10.9	14.9		19.8	23.6	21.7	18.6	23.5	
	Illiterate	6.9	9.9	6.6	8.7	9.6	<0.001	41.0	45.2	38.1	43.5	39.2	<0.001
Housing	Public or aided	37.9	43.8	40.0	42.6	45.1		39.9	41.1	42.2	43.1	37.3	
	Private housing (rent)	6.1	5.8	4.6	4.3	5.3		4.7	5.0	5.0	7.2	2.0	
	Private housing (self-owned flat)	49.8	42.8	51.4	47.9	43.7		47.4	44.1	48.4	43.8	58.8	
	Temporary housing	0.8	1.1	0.6	1.5	1.9		1.0	1.5	0.8	2.0	0.0	
	Institutions	3.4	4.8	1.5	2.0	2.1		4.9	7.4	2.1	1.6	2.0	
	Others	2.1	1.7	1.9	1.7	1.9	<0.001	2.1	1.0	1.6	2.3	0.0	<0.001
Exercise	Never	16.5	15.9	16.6	18.1	31.2		14.2	14.2	16.0	17.0	17.7	
	<30 minutes per day	32.4	34.0	36.0	35.2	31.2		35.8	38.7	37.7	36.0	23.5	
	≥30 minutes per day	51.1	50.1	47.5	46.7	37.6	<0.001	50.0	47.1	46.3	47.1	58.8	0.001
Monthly expenditure	<1000HK$	12.1	12.9	12.2	9.4	9.1		17.2	17.5	14.9	17.7	15.7	
	1000- <2000 HK$	36.1	37.2	37.0	37.9	28.5		39.4	38.5	40.2	38.6	29.4	
	2000- <3000 HK$	30.9	32.0	31.6	32.8	36.5		29.5	28.4	29.1	29.4	21.6	
	3000- <6000 HK$	17.8	15.6	16.1	16.8	22.1		12.4	14.5	14.2	13.1	31.4	
	6000- <10000 HK$	2.3	1.6	2.3	2.4	2.9		1.1	0.8	1.4	1.0	2.0	
	≥10000 HK$	1.0	0.6	0.9	0.6	0.8	<0.001	0.4	0.3	0.3	0.3	0.0	0.002
Smoking status	Never-smoker	53.2	16.5	31.0	27.1	12.5		90.8	64.7	78.0	66.0	58.8	
	Current smoker	16.5	19.0	22.0	31.7	54.4		3.3	5.5	8.5	17.7	23.5	
	Ex-smoker	30.3	64.5	47.0	41.2	33.1	<0.001	5.9	29.7	13.5	16.3	17.7	<0.001
Body mass index	<18.5	6.3	7.0	4.1	5.3	5.1		5.3	5.4	4.7	8.2	9.8	
	18.5-<23	33.7	32.2	32.7	34.8	45.6		30.9	29.9	30.0	33.0	23.5	
	23-<25	24.0	24.1	23.6	23.6	24.0		21.8	21.3	22.1	17.3	23.5	
	25-<30	32.4	32.3	35.4	33.2	20.5		34.7	35.3	34.7	36.0	39.2	
	≥30	3.6	4.4	4.3	3.1	4.8	<0.001	7.3	8.0	8.6	5.6	3.9	0.10

## Results

There were 18,750 men and 37,417 women in the study. After excluding 1951 clients with no follow-up information and 126 clients with missing, relevant baseline data, there were 95.5% (54,090) with follow-up and complete baseline data, including 3819 deaths. There were 188 and 218 deaths from IHD in men and women respectively and 24 and 10 from cancer of the esophagus in men and women. Men were much more likely to use alcohol than women (Table 1). Never-drinkers were much more likely to be never-smokers than current or former alcohol users in both sexes. Ex-drinkers were older in men and women. In men, never and social drinkers were better educated and more likely to live in self-owned accommodation.

In men there was no association between occasional or moderate alcohol use and death from IHD with hazard ratios of almost exactly unity (Table 2) in both models, although male excessive drinkers had a non-significantly lower risk of IHD mortality. However, male excessive drinkers also had an increased risk of cancer of the esophagus (HR 9.42: 95% CI 2.63 to 33.8). In women, because of the low numbers of moderate and excessive drinkers, all current drinkers were combined. In this group of mainly (89%) social drinkers, alcohol use was associated with slightly lower risk of IHD (adjusted HR 0.88, 95% CI 0.51 to 1.53) and had no effect on cancer of the esophagus.

**Table 2:** Hazard ratios with 95% confidence intervals for mortality from IHD and cancer of the esophagus by level of alcohol use in men and women

					Model 1		Model 2	
	Cause of death	Alcohol use	Number of deaths	Years of follow-up	HR	95% CI	HR	95% CI
Men	IHD	Never drinker	92	37,268	1		1	
		Ex- drinker	48	13,228	1.36	0.96 to 1.93	1.33	0.91 to 1.93
		Social Drinker	34	15,899	0.99	0.67 to 1.47	1.02	0.68 to 1.53
		Moderate	12	5,291	1.01	0.55 to 1.84	1.00	0.54 to 1.83
		Excessive	2	1,502	0.62	0.15 to 2.52	0.56	0.14 to 2.29
	Esophagus cancer	Never drinker	9		1		1	
		Ex- drinker	5		1.59	0.53 to 4.77	1.24	0.39 to 3.92
		Social Drinker	2		0.54	0.12 to 2.51	0.52	0.11 to 2.46
		Moderate	4		3.22	0.99 to 10.48	2.91	0.87 to 9.76
		Excessive	4		11.27	3.45 to 36.74	9.42	2.63 to 33.8
Women	IHD	Never drinker	185	129,470	1		1	
		Ex- drinker	19	8,271	1.40	0.87 to 2.25	1.30	0.80 to 2.13
		Current drinker*	14	12,970	0.90	0.52 to 1.55	0.88	0.51 to 1.53
	Esophagus cancer	Never drinker	8		1		1	
		Ex- drinker	1		1.81	0.23 to 14.6	1.07	0.12 to 9.46
		Current drinker*	1		1.34	0.17 to 10.8	1.03	0.12 to 8.71

Model 1 Adjusted for age group.

Model 2 Adjusted for age group, smoking status, education, housing, monthly expenditure, body mass index group and exercise.

* All three current alcohol use groups were combined for women because of the small number of moderate and excessive drinkers in women.

## Discussion

In a large prospective study of older Chinese, we found no overall association between moderate alcohol use and lower mortality from IHD in either men or women, which is somewhat different from the two previous prospective studies of alcohol and IHD mortality in Chinese men. However, one of these studies from four small communities in Shanghai found moderate alcohol use associated both with a 36% reduction in IHD mortality and with a non-significant reduction in mortality from causes where alcohol usually has no effect or monotonically increases risk, [23] such as liver cancer, lung cancer, hepatic cirrhosis and injuries and accidents [29]–[33]. Such systematic effects suggest that some favorable characteristics of the moderate alcohol users may also have been playing a role, and which was not adequately controlled for. The other study did not specifically consider moderate alcohol use and IHD mortality [24], but instead found that moderate alcohol use protected against all cardiovascular mortality, despite the relatively high proportion of hemorrhagic strokes in China [34], for which alcohol would not be expected to be protective [35].

There are several possible explanations for the differences between our study and a large body of evidence showing that moderate alcohol use protects against IHD mortality. First, the period of follow up was relatively short, with correspondingly few deaths, but also less error due to change in alcohol use. Second, the participants were volunteer attendees at a primary care facility, who might be generally healthier with less need of the potential protection offered by alcohol. Third, we distinguished between life-long never-drinkers, ex-drinkers, occasional (or social drinkers) and moderate drinkers, so that the reference group of never-drinkers was not contaminated by “sick quitters” or people who had never used alcohol because of ill-health. Fourth, the association of alcohol use with IHD may vary with age, however most previous studies have found benefits in older people [36]. Fifth, our participants may have been drinking too much or too little alcohol. Given, the generally low level of alcohol use in Hong Kong [18] and the non-significantly protective effect of excessive use, it is most unlikely that our subjects drank too much alcohol. However, we cannot rule out the possibility that they were drinking too little, particularly the women, who were almost entirely social drinkers. Alternatively the alcohol could have been consumed in binge drinking sessions, however that is unusual in Chinese [18] and in older persons [37]. Sixth, our participants may have been using the wrong type of alcohol, as western or grape wine is rarely drunk [18]. However, ethanol is largely responsible for the cardio-protective effects, although there may be some additional protection from some constituents of red wine [38]. Seventh, our findings may be the result of uncontrolled residual confounding, although our findings were the same with adjustment only for age, or with additional adjustment for socio-economic status and lifestyle, which suggests that confounding due to these adjusted factors is not playing a major role.

Nevertheless, despite these limitations it is possible that moderate alcohol use may have less effect on IHD mortality in some settings, perhaps because of role of co-factor, such as folate intake, [39] or because of the relative importance of the different cardio-protective and detrimental aspects of alcohol. Alcohol raises blood pressure [40], [41], but it also raises HDL-cholesterol [42] and possibly improves insulin sensitivity [42]. Depending on the relative importance of these factors to IHD mortality in different settings, it is possible that the effects of alcohol use may differ, with alcohol being most protective in settings where low HDL is the key factor in IHD, and less protective in settings where high blood pressure is the key factor in IHD. There is some preliminary evidence suggesting that HDL-cholesterol may be a less important predictive factor for IHD events in Chinese than in the Framingham cohort, whilst systolic blood pressure may be more important for cardiovascular events in Asians than the Framingham cohort [43], [44]. We do not have HDL-cholesterol for this cohort, so we cannot investigate whether the effects of alcohol varied with HDL-cholesterol and blood pressure.

### Conclusion

In a population-based prospective study of older Chinese people with or without adjustment for multiple confounders, there was no association between moderate alcohol use and lower IHD mortality. This could be a chance association, or it could indicate that moderate alcohol use may not be as protective against IHD some sub-populations, for reasons which need to be clarified.
