# Correction: Moderate Alcohol Use and Mortality from Ischaemic Heart Disease: A Prospective Study in Older Chinese People

**DOI:** 10.1371/annotation/d27238c8-7f54-4bbb-afa4-b99443f1b9f0

**Published:** 2008-06-11

**Authors:** C. Mary Schooling, Wenjie Sun, Sai Yin Ho, Wai Man Chan, May Ked Tham, Kin Sang Ho, Gabriel M. Leung, Tai Hing Lam

The second author's name appears incorrectly. It should be: Wenjie Sun. The correct citation should read: Schooling CM, Sun W, Ho SY, Chan WM, Tham MK, et al. (2008) Moderate Alcohol Use and Mortality from Ischaemic Heart Disease: A Prospective Study in Older Chinese People. PLoS ONE 3(6): e2370. doi: 10.1371/journal.pone.0002370

